# The Physician Fee Schedule Was Not Built for High-Cost Supplies and Equipment

**DOI:** 10.7759/cureus.67931

**Published:** 2024-08-27

**Authors:** David Rasmussen, Amulya Yalamanchili, Bob Tahara, Gerald A Niedzwiecki, Jason McKitrick, Tarita O Thomas

**Affiliations:** 1 Vascular Surgery, The Vascular Care Group, Burlington, USA; 2 Radiation Oncology, Northwestern University Feinberg School of Medicine, Chicago, USA; 3 Vascular Surgery, Allegheny Vein and Vascular, Bradford, USA; 4 Interventional Radiology, Morton Plant Mease Hospitals Advanced Imaging and Interventional Institute, Clearwater, USA; 5 Health Policy, Liberty Partners Group, Washington, DC, USA

**Keywords:** physician fee schedule, medicare reimbursement, health policy and economics, radiation oncology, vascular surgery, interventional radiology, payment system

## Abstract

As reimbursement from the Medicare Physician Fee Schedule (PFS) continues to decline, cuts to practice expense relative value units disproportionately impact office-based interventionalists and private practices that rely on high-cost equipment. For 195 codes, specialties such as radiation oncology, vascular surgery, and interventional radiology are paid at rates less than their direct costs calculated by the Centers for Medicare and Medicaid Services itself. While reimbursement in the office-based setting continues to decline, high-cost hospital settings receive more payment for the same services. This disparity aligns with trends in care moving to the hospital setting and practice consolidation, resulting in increased costs to the healthcare system and decreased access to care. The current PFS is outdated, and the removal of high-cost supplies and equipment from the PFS is a critical step to reform.

## Editorial

The “Physician Fee Schedule (PFS) Reform” has been top-of-mind for healthcare policymakers for the last few years and for good reason. Private practice closures and hospital system consolidation, both of which drive up total costs and limit patients’ access to care, are key reasons for this concern. In May 2024, congressional hearings included a Ways and Means hearing on “The Collapse of Private Practice” and a House Budget Committee entitled “Breaking Up Healthcare Monopolies” [1-2]. The Medicare Payment Advisory Commission (MedPAC) stated in its June 2024 Report to Congress, “The Commission is concerned that ongoing site-of-service payment differentials distort competition and encourage vertical consolidation” [3]. In recent years, Medicare PFS reimbursement has been rapidly declining due to annual cuts to the “conversion factor” as well as ongoing cuts to physician practice expense “relative value units.”

A conversion factor cut is scheduled to take place yet again on January 1, 2025, and likely will be a catalyst for action later this year. It’s critical, however, that policymakers understand that the physicians on the bleeding edge of private practice closures are the office-based interventionalists and that the main source of their reimbursement cuts relates to “practice expense,” not the conversion factor. (Note: relative value units (practice expense, work, and malpractice) times the conversion factor equals Medicare PFS reimbursement.)

Reimbursement cuts to “practice expense” (defined by the Centers for Medicare and Medicaid Services (CMS) as supplies, equipment, nonphysician clinical labor, and overhead costs) have disproportionately hurt those office-based interventional practitioners who predominantly utilize high-tech (high-cost) supplies and equipment to perform critical patient care in the community setting. Focusing on practice expense as part of overall PFS reform not only addresses a key part of the PFS that urgently needs attention, it also offers the most direct avenue towards fundamentally refocusing the PFS on what it originally was established to be: a method of reimbursing physicians for the work they do rather than for the supplies and equipment they use. Importantly, Medicare reimbursement for high-cost supplies and equipment used for interventional services in the hospital is made through a different fee schedule than the PFS, the Hospital Outpatient Prospective Payment System, which is why reimbursement for hospital-based interventional services continues to increase even as office-based reimbursement for interventional services continues to fall.

To better grasp the ineffectiveness of the PFS, the payment system developed and managed by CMS to reimburse healthcare providers, one need only look at reimbursement trends for office-based interventionalists. Budget neutrality, a critical concept in the PFS, dictates that changes to reimbursement rates for any service cannot substantially impact the overall budget for Medicare payments. Consequently, any increase in payments for one service must be offset by reductions in payments for others. For nearly two decades, policymakers have attempted to shore up non-interventional services, which has led to a reduction in the valuation of office-based interventional services utilizing high-cost supplies and equipment as a result of the PFS “budget neutrality” mechanism.

**Figure 1:**
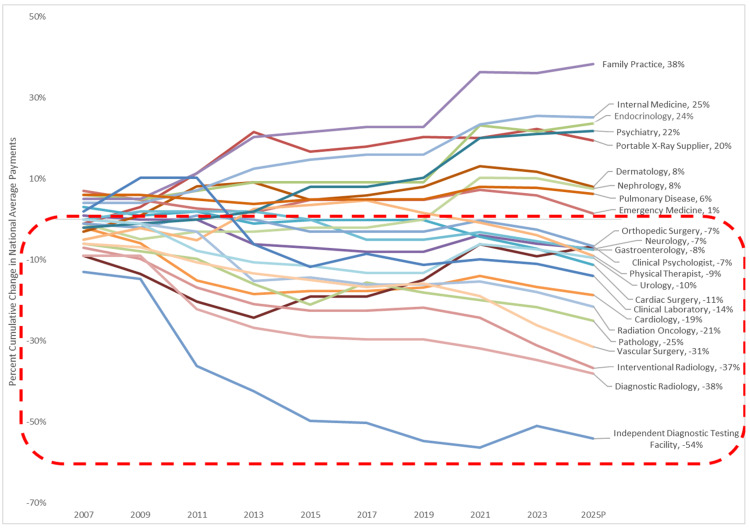
Cumulative impact of changes in RVUs since 2006 The values presented for 2021-2025P (proposed) are adjusted to reflect the effects of the Consolidated Appropriations Act, 2021, 2022, 2023, and 2024. The estimates presented reflect the cumulative impact of changes in relative value units since 2006, as published by CMS in its regulatory impact tables included in the annual PFS rules. The cumulative impact of changes reflects averages at the specialty level and does not account for changes in volume, mix, intensity of services, or site of service over the same timeframe. The cumulative impact at an individual practitioner or practice level may be different from what is presented. The figure was prepared by HMA for the Office-Based Facility Association, of which ACRO is a member. Permission was obtained from ACRO for use; however, inclusion of the figure in the publication does not reflect HMA endorsement of the article content presented. RVUs: Relative Value Units, CMS: Centers for Medicare and Medicaid Services, HMA: Healthcare Management Associates, ACRO: American College of Radiation Oncology, PFS: Physician Fee Schedule Image Source: ACRO Advocacy (2024) [4]

For instance, services like radiation oncology, vascular surgery, and interventional radiology have seen reductions in their service values of over 21% since 2006 (Figure 1). These cuts have become so severe that in the 2024 calendar year, of the base procedures that CMS reimburses in the office, 195 are paid at rates actually less than the direct costs associated with those procedures, as calculated by CMS itself. In the 2025 PFS Proposed Rule released in July, this number would grow to 300, a greater than 50% increase. *In other words, for 300 services, CMS will not pay clinicians in private practice enough to cover the direct expenses of those services before even considering other costs like physician work and indirect expenses.*

**Figure 2:**
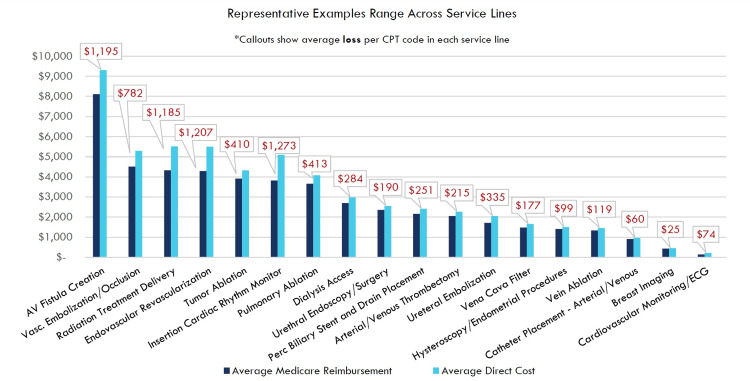
Medicare PFS reimbursement is less than costs for at least 300 office-based interventional services in the PFS 2025 PFS final rule total non-facility reimbursement and total direct costs. Radiation treatment delivery assumes 25 fractions for a typical prostate cancer patient. PFS: Physician Fee Schedule, CPT: Current Procedural Terminology, ECG: electrocardiogram, AV: arteriovenous Image Credit: Author

The services most affected by this inadequate reimbursement are critical for patients and prevent high-cost hospital admissions, ER visits, and death, while also keeping Medicare spending and Medicare beneficiary coinsurance at the lowest cost, office-based site-of-service (Figure 2). For these 300 services, the PFS effectively makes them non-viable in the community-based setting and forces these interventions to migrate back to higher-cost sites of service, such as the hospital-based setting. Such hospital-based outpatient centers provide identical care compared to freestanding centers but are allowed to charge more to offset hospital operational costs. For example, code 36902 for a vascular procedure is billed 343% higher in the hospital setting compared to the outpatient setting for the same exact procedure [3]. Medicare pays out more for the same care in the hospital setting, and patients have decreased access as community practices cannot afford to offer these services.

**Figure 3:**
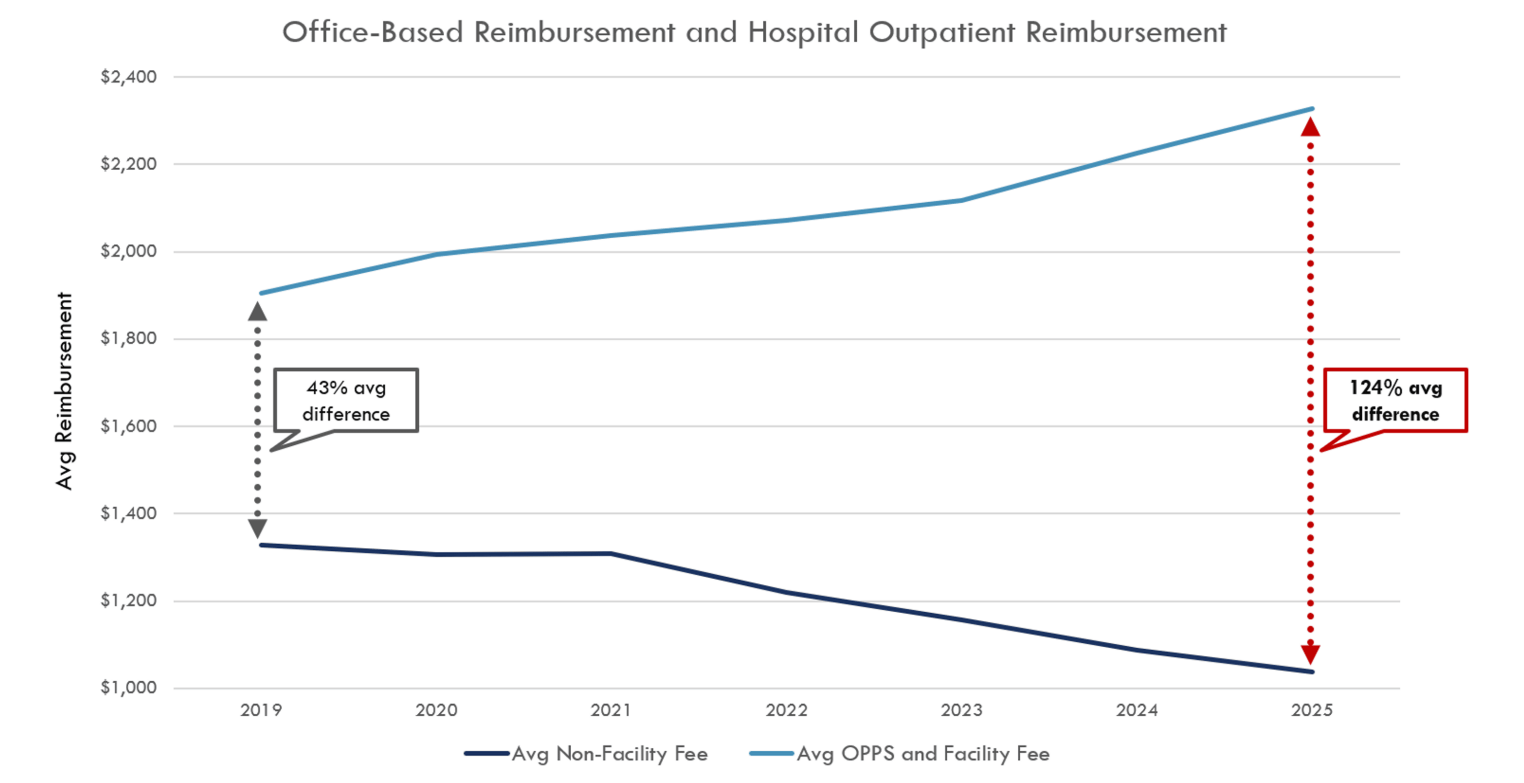
Average office and hospital outpatient reimbursement of 300 underpaid codes Reimbursement is calculated as the average PFS non-facility fee compared to the average PFS facility fee plus the average hospital outpatient department OPPS fee. Two hundred seventy-three of the 300 CPT codes for which total reimbursement is less than direct costs were included. Twenty-seven codes were excluded as they were added to the fee schedule after 2019. PFS: Physician Fee Schedule, CPT: Current Procedural Terminology, OPPS: Outpatient Prospective Payment System Image Credit: Author

Moreover, examining payment trends over recent years for these 300 CPT codes across different sites of service reveals a key catalyst in the increasing consolidation of care. In 2019, the average payment for these codes was reimbursed 34% more when performed in an outpatient hospital setting compared to an office setting. By 2024, this disparity would have ballooned to 93% on average. As the lowest-cost sites of service have faced year-over-year payment reductions, high-cost locations continue to see payment increases (Figure 3). In these interventional specialties, such as radiation oncology and vascular surgery, for example, there has been increased practice consolidation over recent years, with a decrease in office-based Medicare claims and an increase in charges arising from hospital settings [5-6].

This misalignment reflects the reality that when Medicare established the PFS in 1992, policymakers did not anticipate that technology would advance to the point that it would allow minimally invasive endovascular care or radiation therapy to be performed in a community-based setting. These technological advances have been beneficial for patients in terms of lower risk, lower invasiveness, and lower costs, while at the same time increasing both convenience and comfort. However, in a budget-neutral PFS, the migration of more high-tech, high-cost procedures to the office setting adds pressure to a system already struggling to keep pace with rising costs and inflation. Over the last few decades, this significant flaw in Medicare’s fee schedule has diluted the value of all services covered under the PFS, affecting not just office-based interventionalists but also all specialty providers, including those offering predominantly cognitive-based care, such as primary care.

The good news here for policymakers is that focusing on the problem facing community-based interventionalists also reveals the key to reforming the PFS in a fundamentally profound way: policymakers should remove certain high-cost supplies and equipment from the PFS. For well over a decade, the American Medical Association has been asking that “CMS separately identify and pay for high-cost disposable supplies priced more than $500 using appropriate HCPCS codes” (where HCPCS means “healthcare common procedure coding system”) [7]. Certain high-cost equipment, such as equipment used for radiation therapy, does not belong in the PFS. Indeed, it is for this very reason that CMS has not established national rates for a certain subset of radiation therapy called “proton therapy.” Instead, CMS has continued carrier pricing for community-based proton therapy, noting in the 2021 PFS that establishing national rates for proton therapy “at a rate that is so much higher than anything else in our equipment database could distort relativity” [8]. Finally, there is precedent for removing high-cost supplies and equipment from the PFS: in the 2010 PFS, CMS finalized its proposal “to remove physician-administered drugs from the definition of physicians’ services” due to the “significant and disproportionate impact that the inclusion of drugs has had on the SGR (sustainable growth rate) system” [9-10].

In conclusion, today’s PFS is structurally broken, creating barriers to both physician practice viability and patient access to care because it was not established to handle high-technology, high-cost supplies and equipment. By simply removing certain high-cost supplies and equipment from the PFS, policymakers would allow for more appropriate reimbursement for office-based interventional care, mitigate further closure of private practice physicians and preserve access to care, protect the PFS from further dilution, and free up funds for all clinicians as part of overall PFS reform.
